# Clusters of Invasive *Haemophilus influenzae* Type b Disease Among Adults Using Substances or Experiencing Homelessness or Housing Instability — Alaska, Oregon, and Washington, 2023─2025

**DOI:** 10.15585/mmwr.mm7514a1

**Published:** 2026-04-16

**Authors:** Jennifer P. Collins, Heather M. Scobie, Shalabh Sharma, Eric J. Chow, Amy B. Rubis, Tasha Martin, Nicholas R. Graff, Matthew Redlinger, Saba Zewdie, Louisa Castrodale, Julia C. Bennett, George A. Conway, Chas DeBolt, Laurie Orell, Sarah Talia Himmelfarb, Kristen Moore, Ferric C. Fang, Carolynn DeByle, Camerin A. Rencken, Joe McLaughlin, Michael L. Tran, Chi N. Hua, Basanta Wagle, Jennifer Dolan Thomas, LeAnne M. Fox, Jessica R. MacNeil

**Affiliations:** ^1^Division of Bacterial Diseases, National Center for Immunization and Respiratory Diseases, CDC; ^2^Arctic Investigations Program, CDC; ^3^Public Health – Seattle & King County, Seattle, Washington; ^4^Division of Allergy and Infectious Diseases, Department of Medicine, University of Washington, Seattle, Washington; ^5^Department of Epidemiology, University of Washington, Seattle, Washington; ^6^Division of Infectious Diseases, Department of Pediatrics, University of Washington, Seattle, Washington; ^7^Public Health Division, Oregon Health Authority, Portland, Oregon; ^8^Washington State Department of Health, Tumwater, Washington; ^9^Epidemic Intelligence Service, CDC; ^10^Alaska Department of Health, Anchorage, Alaska; ^11^Anchorage Health Department, Anchorage, Alaska; ^12^Harborview Medical Center, University of Washington, Seattle, Washington; ^13^Washington State Public Health Laboratories, Shoreline, Washington.

**Summary**

**What is already known about this topic?**

Since the introduction of *Haemophilus influenzae* type b (Hib) conjugate vaccines in the United States in 1987, invasive Hib disease outbreaks have become uncommon.

**What is added by this report?**

During April 2023–December 2025, two clusters (44 cases) of invasive Hib disease were identified among adults in Alaska, Oregon, and Washington; most patients would not have been eligible for routine Hib conjugate vaccination as children. Smoking (77%), illicit substance use (77%), and housing instability (68%) were common. These clusters demonstrate the vulnerability of adults, particularly those with specific risk factors, to this otherwise rare vaccine-preventable disease.

**What are the implications for public health practice?**

Enhanced surveillance for invasive *H. influenzae* disease in adults could help assess the scope, characterize commonly reported exposures, identify future clusters, and guide development of strategies to protect at-risk populations.

## Abstract

Since the introduction and widespread use of *Haemophilus influenzae* type b (Hib) conjugate vaccines, invasive Hib disease has become rare in the United States, and outbreaks are uncommon. During April 2023–December 2025, two genetically distinct clusters comprising 44 cases of invasive Hib disease in adults were identified: one in Alaska (14 cases) and a second spanning Washington (23) and Oregon (seven). Cases were identified through routine surveillance or notification from a hospital, and clusters were identified via whole-genome sequencing. The median patient age was 53.5 years; 43 (98%) persons had bacteremia and 42 (95%) had pneumonia. Among the 44 patients, 34 (77%) smoked one or more substances, 34 (77%) used illicit substances, and 30 (68%) were experiencing homelessness or housing instability. Overall, 40 (91%) patients did not have documentation of receipt of Hib vaccination; 35 (88%) of those would not have been eligible for routine Hib conjugate vaccination as children because they would have been older than the recommended age range for vaccination at the time the vaccine was introduced. These emerging Hib clusters reveal that adults, particularly those using substances or experiencing homelessness or housing instability, are at risk for this otherwise rare vaccine-preventable disease. Data to guide an optimal public health response to these clusters are limited. Expanding surveillance for invasive *H. influenzae* disease in adults could help assess the scope of this problem, identify future outbreaks, and guide the development and implementation of strategies for prevention.

## Introduction

Before the introduction of *Haemophilus influenzae* type b (Hib) conjugate vaccines in 1987, Hib was a leading cause of invasive bacterial disease (including meningitis, epiglottitis, pneumonia, and septic arthritis) in children aged <5 years (*1*). As a result of the widespread use of Hib vaccines through routine childhood immunization, invasive Hib disease is rare among children and adults, although American Indian and Alaska Native populations are disproportionately affected (*2*). Outbreaks of invasive Hib disease are uncommon (*2*). In British Columbia, Canada, an increase in invasive Hib disease was recently reported among adults, particularly those experiencing homelessness or housing instability and those using substances, including excessive alcohol use (*3*). During April 2023–December 2025, 44 cases of invasive Hib disease in adults were identified in Alaska, Washington, and Oregon through routine surveillance or hospital notification; whole-genome sequencing (WGS) identified two distinct clusters. This report describes the characteristics of these cases and considerations regarding public health response activities.

## Methods

### Data Sources

Invasive *H. influenzae* disease (including Hib) is nationally notifiable, but reporting varies by state based on factors such as patient age or serotype. In Alaska, confirmed cases of invasive Hib disease in adults were identified through routine surveillance, and an increase in Hib cases among adults* was reported to CDC’s Division of Bacterial Diseases in March 2025. In Washington, invasive Hib disease was only reportable in children aged <5 years; confirmed Hib cases among adults were first identified by an academic hospital, which notified the local health department about an increase in invasive *H. influenzae* disease among adults, after which the state health department and CDC were notified. Subsequent serotyping at the state public health laboratory revealed that several cases were caused by Hib. In Oregon, where invasive Hib disease in persons of all ages is reportable, confirmed cases with isolates that were genetically related to Washington’s isolates were identified through routine surveillance via CDC’s Active Bacterial Core surveillance (ABCs).^†^ Patient information, including demographic and clinical characteristics and social risk factor information, was obtained primarily via medical chart review and abstraction.

### Cluster Definition and Inclusion Criteria

A confirmed case of invasive Hib disease was defined as isolation of Hib from a normally sterile body site. Isolates were sent to CDC for molecular typing and WGS analysis. A cluster was defined as two or more confirmed cases of invasive Hib disease with isolates that were closely related based on WGS^§^ (*4*). All cases of invasive Hib disease among adults aged ≥18 years in Alaska, Oregon, and Washington with specimen collection dates during April 2023–December 2025 that met this cluster definition were included in this analysis.

### Analysis

In all three jurisdictions, routine public health investigations were conducted, and a descriptive analysis was performed. This activity was reviewed by CDC, deemed not research, and was conducted consistent with applicable federal law and CDC policy.^¶^

## Results

### Reported Cases and Geographic Distribution

During April 2023–December 2025, a total of 44 cases of invasive Hib disease in adults comprising two genetically distinct clusters were identified: one in Alaska (14 cases) and a second spanning Washington (23) and Oregon (seven)** (Figure). Cases were primarily reported in urban areas; all Alaska cases were reported in Anchorage, all Washington cases were reported in Seattle/King County, and six of seven Oregon cases were reported in the Portland tri-county area.

**FIGURE:**
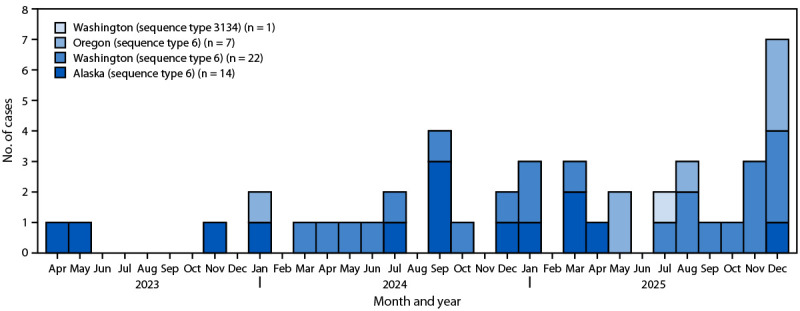
Number and sequence types of invasive *Haemophilus influenzae* type b disease cases in adults, by state, month, and year — Alaska,* Oregon,^†^ and Washington,^§^ April 2023–December 2025 **Abbreviation**: Hib = *Haemophilus influenzae* type b. * All 14 Hib cases in Alaska were reported in Anchorage and were caused by highly related bacterial strains (sequence type 6). ^†^ The seven Hib cases in Oregon were caused by highly related bacterial strains (sequence type 6) that were related to the cases from Washington but not those from Alaska; six cases were reported in the Portland tri-county area. ^§^ All 23 Hib cases in Washington were reported in Seattle/King County. They were caused by highly related bacterial strains (sequence type 6 [22] and sequence type 3134 [one]) that were related to the sequence type 6 cases from Oregon but not those from Alaska.

### Characteristics of Cases

Among all 44 patients, the median age was 53.5 years (range = 19–86 years) and 27 (61%) were male (Table). Twenty-four (55%) patients were White, 12 (27%) were American Indian or Alaska Native, and eight (18%) were Black or African American; all patients were non-Hispanic. Forty-three (98%) patients had bacteremia and 42 (95%) had pneumonia. Forty (91%) patients were hospitalized, and five (11%) died during their illness. Twelve (27%) patients had an underlying lung condition, and six (14%) had an immunocompromising condition. Overall, four (9%) patients had documentation of receipt of Hib vaccination (≥1 dose); of the remaining 40 persons, 35 (88%) would not have been eligible for routine Hib conjugate vaccination as children because they would have been older than the recommended age range for vaccination at the time the vaccine was introduced^††^ (*5*).

**TABLE:** Characteristics of adults with invasive *Haemophilus influenzae* type b disease — Alaska, Oregon, and Washington, April 2023–December 2025

Characteristic	No. (%)
**Total**	**44 (100)**
**Age, yrs, median (range)**	53.5 (19–86)
**Sex**	
Female	17 (39)
Male	27 (61)
**Race and ethnicity**	
White, non-Hispanic	24 (55)
American Indian/Alaska Native, non-Hispanic	12 (27)
Black or African American, non-Hispanic	8 (18)
Clinical syndrome*	
Bacteremia	43 (98)
Pneumonia	42 (95)
Meningitis	1 (2)
Substance use*	
Smoking^†^	34 (77)
Illicit substance use^§^	34 (77)
Injection drug use	9 (20)
Excessive alcohol use	8 (18)
**Other factors**	
Underlying lung condition^¶^	12 (27)
Immunocompromising condition**	6 (14)
Homelessness or housing instability	30 (68)
**Outcome**	
Hospitalized	40 (91)
Survived	39 (89)
Died	5 (11)

* Categories are not mutually exclusive; percentages might sum to >100%.

^†^ Substances smoked included tobacco (27), marijuana (15), fentanyl (13), methamphetamines (seven), and cocaine (three).

^§^ Substances used included amphetamines/methamphetamines (28), opioids (27), and cocaine (nine).

^¶ ^Seven persons had asthma, and seven persons had chronic obstructive pulmonary disease.

** Four persons had HIV infection with a CD4 count <100 cells/mm^3^ at the time of illness, one person had HIV infection with an unknown CD4 count at the time of illness, and one person had cancer.

Homelessness or housing instability was identified among 30 (68%) persons with invasive Hib disease. Overall, 34 (77%) smoked at least one substance, including tobacco (27), marijuana (15), fentanyl (13), methamphetamines (seven), and cocaine (three). Use of illicit substances was identified among 34 (77%) patients; substances reported included amphetamines/methamphetamines (28), opioids (27), and cocaine (nine). Injection drug use was identified among nine (20%) patients and excessive alcohol use among eight (18%).

### Whole-Genome Sequencing

WGS demonstrated that all isolates were sequence type 6 (ST-6) except one isolate from Washington, which was ST-3134. Phylogenomic analysis stratified the isolates into two clusters, which are separated by >200 single nucleotide polymorphisms. One cluster included all isolates from Alaska, and the other included all isolates from Oregon and Washington, including the ST-3134 isolate.

## Discussion

The emergence of clusters of invasive Hib disease among adults, particularly those using substances or experiencing homelessness or housing instability, demonstrates that this population is at risk for an otherwise rare vaccine-preventable disease. Most affected persons would not have been eligible for routine Hib conjugate vaccination as children based on their age, and any potential existing immunity from either natural exposure or vaccination might have waned over time.

Chronic substance use is known to increase the risk for infections through numerous mechanisms, including modulation of host immune responses (*6*). Although relatively few affected persons (14%) had a documented immunocompromising condition, many (77%) had documentation of illicit substance use.^§§^ Similarly, a 2019 meta-analysis found tobacco smoking to be significantly associated with development of community-acquired pneumonia (*7*). The high prevalences of smoking tobacco and other substances (77%) and of pneumonia (95%) among these patients suggest that smoking might be an important contributor. By comparison, among 74 adults with invasive Hib disease in ABCs during 2014–2023,^¶¶^ 37 (50%) had pneumonia.

The occurrence of two distinct ST-6 clusters in the United States, along with multiple Hib sequence types reported in similar populations in British Columbia (*3*), suggests that strain characteristics alone are likely not the primary drivers of these clusters. Additional genomic analyses are needed to better understand the molecular epidemiology of invasive Hib disease, particularly among adults and in these clusters. Both ST-6 and ST-3134 are a part of clonal complex 6. A historic study with limited sample size found clonal complex 6 was responsible for most invasive Hib infections (*8*). CDC did not routinely sequence invasive Hib disease isolates before 2026, which limits the ability to estimate the prevalence of clonal complex 6 in the United States.

Investigating and responding to clusters of invasive Hib disease in adults, particularly those using substances or experiencing homelessness, presents challenges for public health authorities. Identifying possible epidemiologic connections among cases can be limited by difficulty contacting affected persons, transitory and not easily identifiable social networks (e.g., related to drug use), and the potential for Hib to be spread via asymptomatic carriers. Similar clusters might not be detected in jurisdictions where invasive Hib disease among adults is not reportable or where information about risk factors is not collected.

Data needed to guide the development and implementation of an optimal vaccination strategy to control outbreaks or clusters of invasive Hib disease in at-risk adult populations are limited. Small studies suggest that a single dose of Hib conjugate vaccine is immunogenic and well-tolerated in adults who are healthy or who have certain immunocompromising conditions (*9*–*10*). Precedent exists for offering Hib conjugate vaccine to adults to control outbreaks: in response to an outbreak of invasive Hib disease among persons experiencing housing instability in British Columbia, Canada, the local health department implemented an Hib vaccination campaign, which was followed by fewer reported cases. Currently, Hib vaccination is being offered to adults experiencing homelessness in Anchorage (G. Conway, MD, Anchorage Health Department, personal communication, January 30, 2026). Providing Hib vaccination in jurisdictions with larger populations of adults experiencing homelessness would be limited by resource availability and might require further refinement of target populations based on risk factors.

### Limitations

The findings in this report are subject to at least three limitations. First, some cases in these clusters might not have been identified, particularly in Washington where invasive Hib disease was not reportable among adults. Second, because case information was obtained primarily via medical records, the prevalence of substance use and homelessness or housing instability, while high, might be underestimated and some variables might be subject to misclassification. Finally, the epidemiology of these regional clusters might not be nationally representative.

### Implications for Public Health Practice

To assess the scope of invasive Hib disease among adults using substances or experiencing homelessness or housing instability, and to optimize detection of future outbreaks, surveillance for invasive *H. influenzae* disease in adults will need to be enhanced. To address this emerging public health issue, additional investigation is needed to better understand the contribution of poor immunity to Hib, nasopharyngeal colonization with Hib, comorbid health conditions, and social risk factors. State health departments are encouraged to notify CDC via secure email (meningnet@cdc.gov) regarding cases of invasive Hib disease in adults using substances or experiencing homelessness or housing instability or regarding any clusters of invasive Hib disease among adults.

## Footnotes

* The Alaska Department of Health and CDC Arctic Investigations Program maintain statewide laboratory-based surveillance for invasive *H. influenzae* disease in persons of all ages in Alaska.

† Oregon Health Authority conducts statewide, active, laboratory- and population-based surveillance for cases of invasive *H. influenzae* disease in persons of all ages through ABCs. Active Bacterial Core surveillance (ABCs) | CDC

§ WGS was performed using New England Biolabs Ultra II reagents on the Illumina NextSeq platform. Genome characterization was carried out with the Bacterial Meningitis Genomic Analysis Platform. Phylogeny was created using a standard outbreak pipeline. Whole-genome alignment was performed using Snippy v4.6.0. Recombination correction was conducted using Gubbins v2.4.1. Final phylogeny was created using RaxML v8.2.12. Phylogeny annotation and visualization were created using iTOL (version 7.5.1; European Molecular Biology Laboratory). Whole genome single nucleotide polymorphisms (SNP) analysis was carried out using kSNP3 v3.1.0. The average SNP distance between isolates was 8 SNPs in the Alaska clade and 3 SNPs in the Washington and Oregon clade.

¶ 45 C.F.R. part 46, 21 C.F.R. part 56; 42 U.S.C. Sect. 241(d); 5 U.S.C. Sect. 552a; 44 U.S.C. Sect. 3501 et seq.

** One additional case of invasive Hib disease in an adult (from Oregon) was identified during the study period and was excluded because it did not meet the cluster definition.

†† Persons born before January 1986 were considered ineligible for routine Hib conjugate vaccination as children, based on first licensure and recommendation of a single dose for children aged 18─23 months in December 1987.

§§ By comparison, the 2024 National Survey on Drug Use and Health found 24.8% of U.S. adults aged ≥26 years reported illicit drug use in the past year, inclusive of marijuana use (21.7%), and exclusive of illegally manufactured fentanyl. However, because all three jurisdictions have laws permitting recreational use in adults, this report does not classify marijuana as an illicit drug.

¶¶ 2014–2023 are the most recent 10 years of finalized ABCs data.
